# Time to Include Vestibular Neurology as a Core Competency for Neurology Trainees

**DOI:** 10.1212/NE9.0000000000200257

**Published:** 2025-09-30

**Authors:** Barry M. Seemungal, Hena Ahmad, Sergio Carmona, Michele Corrado, Caroline Froment Tilikete, Natasha Harrington-Benton, Christoph Helmchen, Diego Kaski, Ji-Soo Kim, Amir Kheradmand, Ashni Khetarpal, Sudhir Kothari, Heiko M. Rust, Cynthia Ryan, Aasef G. Shaikh, Miriam S. Welgampola, Andreas Zwergal, Alexander A. Tarnutzer

**Affiliations:** 1 Centre of Vestibular Neurology, Department of Brain Sciences, Imperial College London, United Kingdom;; 2 Department of Neurology, Atkinson Morley Regional Neuroscience Centre, St George's University Hospitals NHS Foundation Trust, London, United Kingdom;; 3 Buenos Aires Institute of Neuroscience (INEBA), Buenos Aires, Argentina;; 4 Headache, Science and Neurorehabilitation Centre, IRCCS Mondino Foundation, Pavia, Italy;; 5 CRNL, équipe Impact, Université Lyon 1, Faculté de Médecine Lyon Est, Hospices Civils de Lyon, Service de Neuro-ophtalmologie et Neurocognition, France;; 6 The Meniere's Society, United Kingdom;; 7 Department of Neurology, University Hospital Schleswig Holstein; Campus Lübeck, Germany;; 8 Department of Neurology, National Hospital for Neurology and Neurosurgery, London, United Kingdom;; 9 Department of Neurology, Seoul National University College of Medicine, Seoul National University Bundang Hospital, South Korea;; 10 Amir Kheradmand, Departments of Neurology, Neuroscience, Otolaryngology–Head & Neck Surgery, and Laboratory for Computational Sensing and Robotics (LCSR), Johns Hopkins Hospital, Baltimore;; 11 Association of British Neurologists Trainees Chair and Imperial College Healthcare NHS Trust, London, United Kingdom;; 12 Poona Hospital and Research Centre, Pune, India;; 13 Department of Neurology, University of Basel Hospital, Switzerland;; 14 Department of Neurology, University Hospitals, Case Western Reserve University, Cleveland, OH;; 15 The Vestibular Disorders Association (VeDA);; 16 Faculty of Medicine and Health Royal Prince Alfred Hospital, University of Sydney, Australia;; 17 Department of Neurology & German Center for Vertigo and Balance Disorders (DSGZ), LMU University Hospital, LMU Munich, Germany; and; 18 Neurology, Cantonal Hospital of Baden, 5404 Baden, Switzerland and Faculty of Medicine, University of Zurich, Switzerland.

Murray noted^1^ health care delivery and hence training curricula should align with patients' needs. Although proportions vary by region, the most common presentations in general neurology are headache, dizziness, and fits or faints.^2^

Although headache and seizures are core neurology curriculum topics, vestibular neurology is not consistently recognized as a core competency worldwide. This results in a mismatch between patient needs and training expectations.

Historically, suppurative inner-ear disease was the most common cause of vertigo and deafness, becoming rare with widespread use of antibiotics and vaccinations. In developed and developing countries, 3 conditions are responsible for 60% of vestibular presentations.^3^ This includes vestibular migraine (VM; 1-year population prevalence, 3%), benign paroxysmal positional vertigo (BPPV; 1-year population prevalence, 1.6%), and orthostatic hypotension (OH; 22%-point prevalence >65 years). Patients frequently present with subjective symptoms, which complicates diagnostic certainty. As Murray^1^ argued, neurologic teaching should prioritize the approach to undifferentiated problems rather than focusing solely on established diagnoses. Conditions such as VM, BPPV, and OH can all produce overlapping symptoms of dizziness, prompting referrals variously to ENT (otolaryngology) neurology, or cardiology. As a result, patients are frequently passed between body part-specific clinics and left with ambiguous labels like “not a brain problem” or “not an ear problem”. Delay in diagnosis can lead to development of chronic functional vestibular syndromes (persistent postural perceptual dizziness [PPPD]).

Stroke misdiagnosis, which is twice as likely for stroke with vertigo, is the largest preventable cause of death or disability from misdiagnosis in the United States (∼795,000/y).^4^ In acute vestibular syndrome (AVS) with vertigo and imbalance, clinicians sometimes apply a binary classification: stroke vs nonstroke. Brain MRI is falsely negative in 20% of stroke AVS.^5^ A trained clinician's bedside vestibular assessment outperforms neuroimaging and assessments by untrained clinicians, enabling gold standard diagnosis and management of AVS.^6^

Vestibular neurologic skills are widely applicable across several areas of neurology and enhance diagnostic precision when used to understand the wider set of vestibular diagnoses. Such conditions include downbeat nystagmus, bilateral peripheral vestibular failure, neurodegeneration (e.g., tauopathies with falls and early oculomotor signs), imbalance or ocular dysmotility in multiple sclerosis or brainstem neuroinflammation (e.g., periodic alternating/pendular nystagmus), and combined central-peripheral dysfunction in “*cerebellar ataxia, vestibular areflexia syndrome*,*”* or traumatic brain injury, often with vestibular agnosia.^7^

Vestibular neurologic training enhances managing common and complex presentations like elderly patients with repeated falls from multiple diagnoses or multimorbidities. These may include BPPV-*sans*-vertigo, autonomic neuropathy, proximal myopathy, small vessel disease–related imbalance, and “PPPD” from prior inadequate management (often from underappreciated complexity).

The field of vestibular neurology has benefitted from advances in genomic medicine which has improved diagnostic assessment, monitoring, and treatment (e.g., imbalance from the highly prevalent GAA-FGF14/spinocerebellar-ataxia-27B8 responds to aminopyridines^8^).

A neurology curriculum aligned with patients' needs requires high competency in common office and acute vestibular syndromes. A 3-4-month clinical experience with expert supervision and personal study (including neuroanatomy, physiology, molecular genetics) should ensure the trainee can identify, interpret, and understand vestibular symptoms, signs, and investigations (e.g., video head-impulse, caloric-irrigation, video-oculography).

Competence in subspecialty skills in vestibular neurology is typically acquired during doctoral fellowship training. Detailed description of these is outside of the scope of this article; however, subspecialists are required as trainers and supervisors. The Bárány society is developing a relevant comprehensive vestibular training curriculum (due to launch 2026).

Implementing upgraded curricula will require a transitional phase. During this time, the shortage of trained supervisors and the recognition of unmet patient needs are likely to encourage growth in vestibular neurology subspecialization. Ultimately, health services must adapt to evolving patients' needs and new treatment opportunities. Recognizing vestibular neurology as a core neurologic competency for neurology trainees is urgently needed to improve care for a large proportion of our patients.
